# Improving Antiemetic and Analgesic Prescribing Practices for Patients With Advanced Liver Disease: A Clinical Audit

**DOI:** 10.7759/cureus.79920

**Published:** 2025-03-02

**Authors:** David Baird, Scott Calvert, Fiona Finlay

**Affiliations:** 1 Gastroenterology and Hepatology, NHS Greater Glasgow and Clyde, Glasgow, GBR; 2 Palliative Care, NHS Greater Glasgow and Clyde, Glasgow, GBR

**Keywords:** : analgesia, antiemetic agents, clincal audit, decompensated liver failure, guideline, hepatology, supportive and palliative care

## Abstract

Background and objective

Previous studies have demonstrated that local analgesic and antiemetic prescribing practices were inconsistent with best practices. In light of this, we aimed to assess the current prescribing practices against best practices, as defined by national guidelines.

Methods

This study involved a point-prevalence audit in the inpatient gastroenterology wards at a University Teaching Hospital in Glasgow. Patients were identified by case notes; a clinical diagnosis of cirrhosis documented by a consultant hepatologist was required for inclusion. Data including Child-Pugh score and prescribed medications were collected. Prescribing practice was reaudited after each intervention. Interventions included educational seminars, posters, and the formulation of new local guidelines.

Results

A total of 249 inpatients were included; 70 were identified as having a clinical diagnosis of cirrhosis. The number of patients who were prescribed weak opioids decreased from 23% in cycle 1 to 11% in cycle 3. Prescription of 3 g/day paracetamol decreased from 36% to 24%. The number of patients who were prescribed an inappropriate antiemetic decreased from 45% to 17%. Laxatives, topical analgesics, and adjuvant analgesics were variably prescribed in all three cycles.

Conclusions

The development of concise local guidelines and targeted educational interventions for junior medical staff can improve prescribing practices for inpatients with decompensated cirrhosis. Currently, significant variability exists in the routine prescription of laxatives, adjuvants, and topical analgesics.

## Introduction

Rational prescribing for patients with advanced liver disease is challenging. Cirrhosis affects the pharmacokinetics of many drugs, leading to unpredictable therapeutic and adverse effects. In addition, this patient group has a high burden of symptoms and is vulnerable to side effects such as constipation and sedation, which may precipitate hepatic encephalopathy [1]. Analgesics and antiemetics are often prescribed by junior members of the medical team, out-of-hours, and without senior advice. This highlights the need for some improvement in prescribing habits with targeted educational interventions. We aimed to compare antiemetic and analgesic prescribing practices for patients with cirrhosis with best practices, as outlined in the Scottish Palliative Care Guidelines (SPCG) for end-stage liver disease. We aimed to improve compliance with best practices. In particular, we aimed to decrease inappropriate antiemetic prescriptions, decrease paracetamol prescriptions of >3 g in 24 hours, and increase the proportion of patients who are co-prescribed laxatives.

Non-opioid analgesics

Paracetamol is a safe, effective and opioid-sparing analgesic. It undergoes hepatic metabolism via conjugation with glutathione. States of glutathione depletion - such as cirrhosis, chronic alcohol use, and malnutrition - may increase the risk of hepatotoxicity through the generation of N-acetyl-p-benzoquinone imine (NAPQI) [1]. However, hepatotoxicity with therapeutic doses of paracetamol has not been observed in patients with significant risk factors for glutathione depletion [2]. Hence, significant heterogeneity exists between guidelines. The SPCG recommends a standard dose of 1 g four times a day for patients with a "normal lean body weight", though this may be challenging to estimate in patients with refractory ascites [3]. It also recommends a dose reduction in patients 50 kg in weight, and those with poor nutritional status and severe hepatic impairment [3].

The British Association for Study of the Liver (BASL) stratifies paracetamol dosing by duration, with a dose of 2-3 g per 24 hours recommended for treatment durations of seven days, and 1 g four times a day for treatment durations less than or equal to seven days [4]. Previous retrospective studies have suggested that patients with cirrhosis are more likely to be started on an opioid due to the perceived risks of non-opioid analgesia [5]. Non-topical non-steroidal anti-inflammatory drugs (NSAIDs) are contraindicated in patients with advanced liver disease due to an increased risk of gastrointestinal bleeding and hepatorenal syndrome. In addition, most NSAIDs are protein-bound and their free-plasma concentrations may be increased due to hypoalbuminemia [6]. Limited evidence suggests that topical analgesics may be effective for musculoskeletal pain, and are associated with low rates of systemic adverse events [7].

Opioid analgesics

Cirrhosis changes the metabolism of opioids via several mechanisms, including reduced first-pass metabolism, hypoalbuminemia, cachexia, and cholestasis. Codeine is the most frequently prescribed opioid in the UK [8]. However, there is considerable variability in its effects due to metabolism by the CP450 enzyme system to morphine. Extensive metabolisers may be exquisitely sensitive to codeine, while poor metabolisers may incur limited therapeutic benefits [9]. This variability is increased by hepatocellular dysfunction in cirrhosis. For this reason, the use of codeine and dihydrocodeine in advanced liver disease is not recommended by SPCG or BASL [3,4]. Morphine is the prototypical opioid and is widely used for inpatient analgesia in the UK. *In vivo *studies of morphine pharmacokinetics in patients with cirrhosis have shown a significantly higher oral bioavailability and longer plasma half-life [10]. Therefore, it is recommended that morphine be dosed cautiously in patients with cirrhosis and titrated to effect, so as not to precipitate hepatic encephalopathy. Similarly, other strong opioids, such as oxycodone, alfentanil, and fentanyl are largely safe to use in cirrhosis with careful titration of dosages [3,4].

Antiemetics

Alongside pain, nausea and vomiting significant causes of distress and decreased quality of life among patients with advanced liver disease [11]. The etiology of nausea and vomiting in cirrhosis is complex and may be related to varices, gut mucosal changes, and the pressure effects of abdominal ascites [12]. Patients with cirrhosis may have increased caloric requirements due to hypermetabolism. Moreover, protein-calorie malnutrition has been associated with increased in-hospital mortality [13]. This makes effective antiemetic therapy an important clinical priority. Dopamine receptor antagonists, such as metoclopramide and domperidone, are recommended as first-line antiemetics by both SPCG and BASL [3,4]. Uniquely, these drugs are pro-kinetic and may increase lower oesophageal sphincter tone. Ondansetron, a 5-HT3 antagonist, is licensed in the UK for chemotherapy-induced and postoperative nausea and vomiting [14]. It is considerably associated with constipation and is recommended as a second-line drug by BASL and SPCG. Cyclizine is associated with both sedative effects and constipation and is recommended as a third-line medication by BASL and SPCG [3,4].

## Materials and methods

All inpatients admitted to our tertiary centre's three adult gastroenterology wards were included in a single-day point prevalence audit. A total of 84 patients were screened each cycle. Inclusion criteria were a diagnosis of cirrhosis of any etiology. This was defined as a clinical diagnosis made by a gastroenterologist and documented in patient notes. Patients without a formal diagnosis of cirrhosis were excluded.

We used an audit tool to collect demographic data, Child-Pugh scores, and prescribed medications of interest. Medications of interest included paracetamol, non-topical NSAIDs, weak opioids, strong opioids, laxatives, topical agents, and adjuvants. Weak opioids were defined as codeine, dihydrocodeine, tramadol, co-codamol, and co-dydramol. Strong opioids were defined as morphine, oxycodone, and alfentanil, as well as non-topical preparations of fentanyl and buprenorphine. Topical agents were defined as lidocaine patches and menthol cream. Adjuvant medications were defined as gabapentin, pregabalin, amitriptyline, and clonazepam. 

We further stratified paracetamol by prescribed dose. Due to the ambiguity of SPCG guidelines for paracetamol, we used 3 g/day as our standard dosage, judging that most patients requiring admission for decompensated cirrhosis fulfilled the criteria of "concern about nutritional status" requiring dose reduction. We defined "inappropriate antiemetic" as the first-line prescription of cyclizine or ondansetron based on SPCG guidelines. Domperidone, an alternative first-line antiemetic, is not routinely used at our centre.

**Figure 1:**
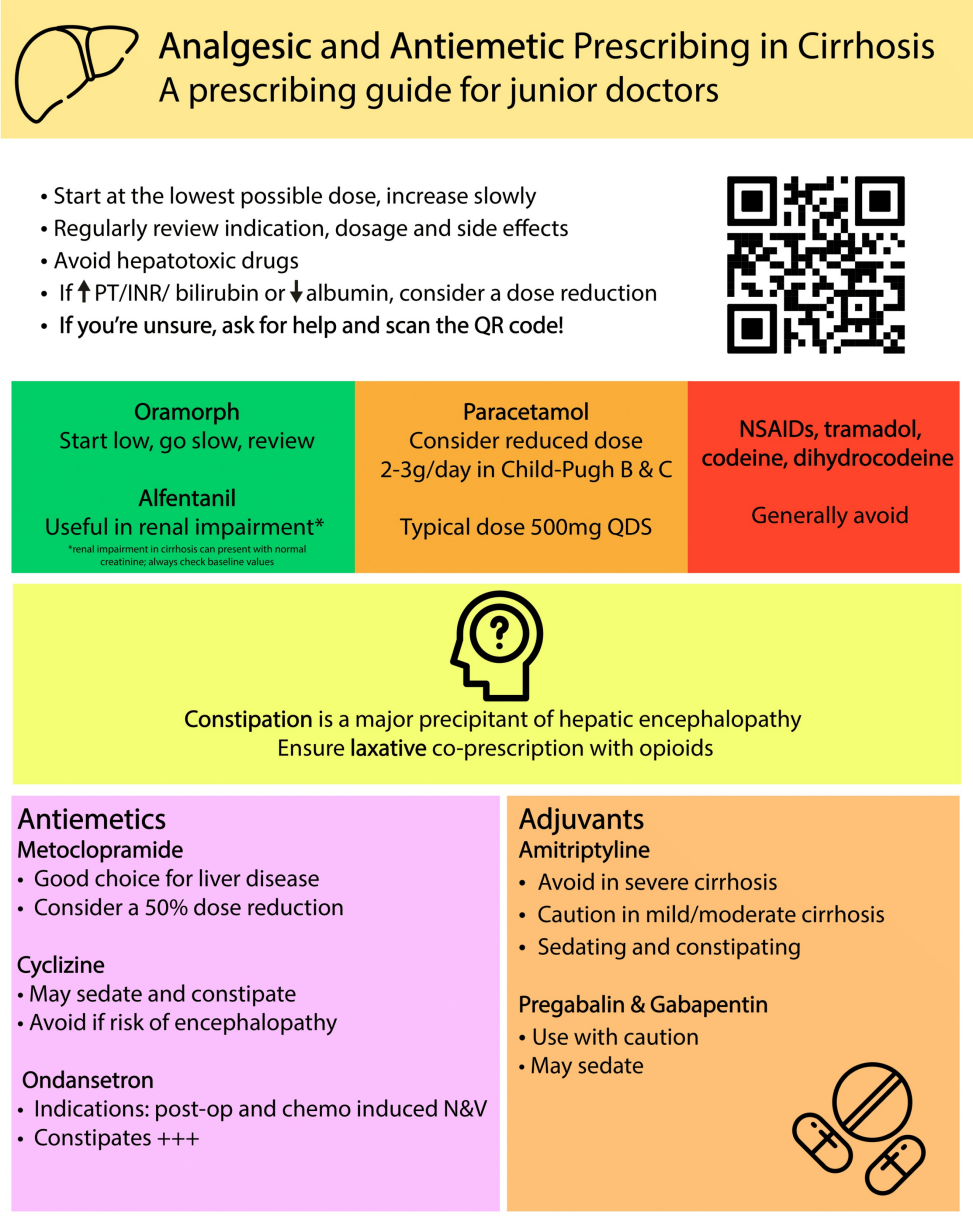
An example of one of our educational interventions This poster was displayed prominently in three inpatient gastroenterology wards

We conducted three cycles and implemented two educational interventions. We used a plan-do-study-act (PDSA) protocol for the clinical audit. An educational intervention was delivered to junior doctors at a departmental meeting in February 2022 based on best practices as defined by SPCG. Educational posters on rational prescribing were displayed prominently in three inpatient gastroenterology wards (Figure 1). These posters emphasised visual appeal and ease of use. We re-audited prescribing in March 2022. SPCG was chosen as our standard, as it is the most used and widely accessible guideline at our centre.

Subsequently, a guideline for analgesic and antiemetic prescribing for inpatients with decompensated cirrhosis was developed and disseminated to clinical staff at our health board in March 2023 [15]. This was based on existing SPCG guidelines and approved by our local Therapeutics Handbook Editorial Group. We then undertook a third audit cycle in August 2023. Numbers of patients for relevant outcomes were tabulated. Rates were expressed as a proportion of total patients in each cycle and subgroup. No formal statistical testing was conducted.

## Results

A total of 84 patients across three gastroenterology wards were screened in each cycle. Of these, 22 patients in cycle 1, 20 patients in cycle 2, and 28 patients in cycle 3 were identified as having cirrhosis. Most patients were male in all three cycles. The most common etiology identified was alcohol-related liver disease (ArLD), followed by metabolic-associated steatotic liver disease (MASLD); metabolic and cryptogenic etiologies were identified in a minority of patients. Most patients had Childs-Pugh B or C cirrhosis (cycle 1: 95%, cycle 2: 85%, cycle 3: 78%).

**Table 1:** Results of successive cycles ^1^Codeine, dihydrocodeine, tramadol, co-codamol, co-dydramol. ^2^Morphine, oxycodone, alfentanil, non-topical preparations of fentanyl and buprenorphine. ^3^Amitriptyline, gabapentin, pregabalin, clonazepam. ^4^Cyclizine, ondansetron prescribed first-line NSAID: non-steroidal anti-inflammatory drug

Drug of interest	Cycle 1 - number (proportion)	Cycle 2 - number (proportion)	Cycle 3 - number (proportion)
Laxative	19/22 (0.86)	14/20 (0.7)	21/28 (0.75)
Non-topical NSAID	0/22 (0.00)	1/20 (0.05)	0/28 (0.00)
Weak opioid^1^	5/22 (0.23)	1/20 (0.05)	3/28 (0.11)
Strong opioid^2^	12/22 (0.55)	10/20 (0.50)	14/28 (0.50)
Topical preparation	1/22 (0.05)	1/20 (0.05)	3/28 (0.11)
Adjuvant analgesic^3^	2/22 (0.09)	1/20 (0.05)	3/28 (0.11)
Paracetamol <3 g/day	7/11 (0.64)	8/10 (0.8)	13/17 (0.76)
Paracetamol >3 g/day	4/11 (0.36)	2/10 (0.20)	4/17 (0.24)
Inappropriate antiemetic^4^	5/11 (0.45)	3/8 (0.38)	2/12 (0.17)

## Discussion

Prescriptions of inappropriate dosages of paracetamol, weak opioids, and antiemetics decreased after educational interventions. These trends were consistent over the 18-month duration of our audit cycle. This suggests that the development of concise, realistic, and intuitive local guidelines for junior medical staff can materially improve prescribing practice in high-risk inpatients. Targeted educational interventions may be of use in other contexts, such as rationalising the use of nephrotoxins and high-risk antibiotics for patients with cirrhosis. 

Despite this, our data showed considerable variability in laxative co-prescription and low utilisation of both adjuvants and topical agents. Low utilisation of topical agents may be due to clinician unfamiliarity, perceived ineffectiveness, or concerns over altered skin perfusion in cirrhosis. In addition, we did not collect data on the type of pain being treated. It may be that these agents are useful in patients with localised neuropathic pain but less so in patients with nociceptive or mixed pain. Topical analgesics may be an effective means of avoiding systemic drug exposure in this patient population.

In addition, variability in the prescription of laxatives may be attributed to patient comfort or preference. Being a single-day point prevalence study, our data may not represent the true prescribing trends over time. In addition, our data captured several patients with Childs-Pugh A cirrhosis. These patients were often hospitalised for reasons other than decompensated cirrhosis. SPCG is intended for patients with Child-Pugh B and C disease. Conversely, our audit also captured a minority of patients receiving care in the last days and hours of life. Prescribing for these patients was likely undertaken by a senior member of medical staff or our centre’s dedicated Hospital Palliative Care Team. Prescribing in these patients was highly compliant with guidelines and may not reflect the typical practice in our unit.

## Conclusions

We observed a notable reduction in the prescription of inappropriate analgesic and antiemetic agents after the implementation of this project. This reduction was sustained over an 18-month period. This highlights the utility of targeted educational interventions among junior doctors, the use of accessible posters, and the need for the development of intuitive regional guidelines.
